# A low‐intensity 10‐min resistance exercise program that ameliorated hepatic fibrosis indices and altered G‐CSF/IP‐10/PDGF‐BB in a patient with nonalcoholic fatty liver disease: A case report

**DOI:** 10.1002/jgh3.12876

**Published:** 2023-02-20

**Authors:** Ryuki Hashida, Dan Nakano, Hiroo Matsuse, Sachiyo Yoshio, Tsubasa Tsutsumi, Machiko Kawaguchi, Shunji Koya, Keisuke Hirota, Hiroshi Tajima, Yoshio Sumida, Tatsuya Kanto, Takumi Kawaguchi, Koji Hiraoka

**Affiliations:** ^1^ Department of Orthopedics, School of Medicine Kurume University Kurume Japan; ^2^ Division of Rehabilitation Kurume University Hospital Kurume Japan; ^3^ Division of Gastroenterology, Department of Medicine Kurume University School of Medicine Kurume Japan; ^4^ Department of Liver Disease, The Research Center for Hepatitis and Immunology National Center for Global Health and Medicine Ichikawa Japan; ^5^ Division of Hepatology and Pancreatology, Department of Internal Medicine Aichi Medical University Nagakute Japan

**Keywords:** cytokines, hepatic fibrosis, inflammation, nonalcoholic fatty liver disease, resistance exercise

## Abstract

We developed a low‐intensity 10‐min resistance exercise program for nonalcoholic fatty liver disease (NAFLD). We report a case of NAFLD with elevated hepatic fibrosis indices, which were improved by a 60‐week daily exercise program. A 71‐year‐old female patient with NAFLD whose hepatic fibrosis stage corresponded to F2 was referred to our hospital. She performed the exercise once a day with no changes in other lifestyle habits and medications. The homeostasis model assessment‐insulin resistance value and NAFLD‐liver fat score, the Hepamet fibrosis score, and the enhanced liver fibrosis score decreased. The FIB‐4 index and serum levels of Mac‐2 binding protein glycosylation isomer decreased to the reference values. We investigated the changes in chemokines/cytokines. The serum granulocyte‐colony stimulating factor level was increased, and serum interferon‐gamma‐induced protein‐10 and platelet‐derived growth factor‐BB levels were decreased. Our program may be beneficial for improving hepatic fibrosis in patients with NAFLD.

## Graphical abstract

Exercise is a first‐line therapy for nonalcoholic fatty liver disease (NAFLD). We developed a low‐intensity 10‐min resistance exercise program, which consists of six types of exercise (three metabolic equivalents). Our exercise program improved hepatic fibrosis in patients with NAFLD.

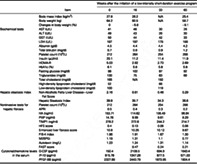

## Introduction

Exercise is the first‐line therapy for various metabolic diseases, including nonalcoholic fatty liver disease (NAFLD). High‐intensity exercise is effective in improving hepatic fibrosis,^1^ which is a prognostic factor in patients with NAFLD. However, evidence regarding the effect of low‐intensity short‐duration exercise on substantial hepatic fibrosis is still lacking.

Exercise can be classified as aerobic and resistance. Resistance exercise requires less energy than aerobic exercise.^2^ Aerobic exercise has the disadvantages of causing fatigue and discomfort, leading to poor long‐term compliance in patients with NAFLD. Thus, resistance exercise may be effective for patients with NAFLD, particularly elderly individuals with comorbidities.^2^ We previously performed a systematic review of exercise therapy for NAFLD^2^ and developed a low‐intensity short‐duration resistance exercise program called Hepatocise.^3^ This program comprises six types of exercise that can be performed in a small space with no special equipment. The exercise requires approximately 10 min per session and is feasible even for individuals with no exercise habits.^3^

For the assessment of hepatic fibrosis, noninvasive assessments are widely used in clinical practice. Transient elastography (FibroScan) is a well‐established imaging method. Additionally, various indices or biochemical parameters are available, including the FIB‐4 index, aspartate aminotransferase to platelet ratio index (APRI), M2BPGi, autotaxin, enhanced liver fibrosis (ELF) score, and Hepamet fibrosis score (HFS). Thus, various indices are currently available. However, it is unclear which index is useful for evaluating treatment response.

Exercise confers health benefits via various mechanisms. Exercise increases energy consumption and improves insulin resistance, a risk factor for hepatic fibrosis. Additionally, exercise has been reported to influence the expression of various chemokines/cytokines. Exercise reduces inflammation‐related biomarkers, including interferon‐gamma‐induced protein‐10 (IP‐10), in healthy adults.^4^ Furthermore, exercise reportedly improved platelet‐derived growth factor‐BB (PDGF‐BB) in obese mice.^5^ Mechanical stress promotes granulocyte‐colony stimulating factor (G‐CSF) secretion from skeletal muscle cells.^6^ These chemokines and cytokines are involved in hepatic fibrosis regulation. However, the effects of low‐intensity exercise on these chemokines/cytokines in patients with NAFLD remain unclear.

Here we report a case of NAFLD with elevated hepatic fibrosis indices, which were improved by implementing just a low‐intensity 10‐min resistance exercise program once daily for 60 weeks. We also show that our exercise program affected several chemokines/cytokines that regulate hepatic fibrosis.

## Case report

A 71‐year‐old Japanese woman was referred to our hospital for NAFLD management. She was treated for hypertension and hyperlipidemia. She had exercised regularly and played table tennis for a long time. However, at 69 years of age, she developed an orthopedic disease in her knees. Since then, she could not play table tennis and developed NAFLD.

The patient underwent cholecystectomy for gallstones when she was 55 years old. Her father also had type 2 diabetes mellitus. The patient characteristics are summarized in Table 1. Physical examination revealed the following: height, 152 cm; weight, 64.3 kg; and body mass index (BMI), 27.8. Her serum aspartate aminotransferase, alanine aminotransferase, and gamma‐glutamyl transpeptidase (GGT) levels were above the reference values. Her total bilirubin and total protein levels were within reference limits. The HbA1c and homeostasis model assessment‐insulin resistance (HOMA‐IR) values were 6.1% and 5.03%, respectively, indicating prediabetes with insulin resistance. There was no evidence of viral hepatitis, autoimmune hepatitis, Wilson's disease, or hemochromatosis.

**Table 1:** Changes in results of body mass index, biochemical test, noninvasive tests for hepatic fibrosis, noninvasive test outcomes for hepatic fibrosis, and cytokines/chemokine levels in the serum after the initiation of a low‐intensity short duration exercise program

		Weeks after the initiation of a low‐intensity short‐duration exercise program			
	Item	0	16	33	60
	Body mass index (kg/m^2^)	27.8	26.2	N/A	25.4
	Body weight (kg)	64.2	60.5	N/A	58.7
	Changes in body weight (%)	0	−5.8		−9.1
Biochemical tests	AST (U/L)	41	46	30	26
	ALT (U/L)	49	43	26	30
	GGT (IU/L)	50	44	42	33
	LDH (U/L)	187	197	179	168
	Albumin (g/dl)	4.5	4.4	4.4	4.2
	Total bilirubin (mg/dl)	0.7	0.9	0.9	1.2
	Platelet count (10^9^/L)	212	264	254	266
	Insulin (μU/ml)	20.1	11.2	11.4	11.9
	HOMA‐R	5.03	2.82	2.73	2.69
	HbA1c (%)	6.1	5.8	5.8	5.8
	Fasting glucose (mg/dl)	98	102	97	92
	Triglycerides (mg/dl)	130	75	63	66
	Total cholesterol (mg/dl)	165	N/A	192	
	High‐density lipoprotein cholesterol (mg/dl)	52		66	
	Low‐density lipoprotein cholesterol (mg/dl)	100		119	
Hepatic steatosis index	Non‐Alcoholic Fatty Liver Disease—Liver Fat Score	2.16	0.81	0.46	0.29
	Hepatic Steatosis Index	39.8	35.7	34.3	36.6
Noninvasive tests for hepatic fibrosis	Platelet count (10^9^/L)	212	264	254	266
	APRI	0.5	0.4	0.3	0.2
	HA (ng/ml)	153.71	114.62	106.49	96.94
	PIIIP (ng/ml)	14.78	9.99	9.61	8.29
	TIMP1 (ng/ml)	278.2	272.6	244.2	214.7
	HFS score	0.4	0.13	0.06	0.06
	Enhanced liver fibrosis score	10.8	10.25	10.12	9.87
	FIB‐4 index	1.96	1.91	1.67	1.30
	M2BPGi	1.19	1.31	1.1	0.77
	Autotaxin (mg/L)	1.23	1.24	1.31	1.14
	FAST score		0.47	0.24	0.21
Cytokine/chemokine levels in the serum	G‐CSF (pg/ml)	150.4	2108.5	694.9	1643.4
	IP‐10 (pg/ml)	915.78	1347.28	877.5	571.03
	PFGF‐BB (pg/ml)	2327.89	2443.79	1873.95	1804.4

Abbreviations: ALT, alanine aminotransferase; APRI, AST to platelet ratio index; AST, aminotransferase; FAST score, FibroScan‐AST (FAST) score; Fib‐4 index, Fibrosis‐4 index; G‐CSF, granulocyte‐colony stimulating factor; GGT, gamma‐glutamyl transpeptidase; HA, hyaluronic acid; HFS, Hepamet fibrosis score; HOMA‐IR, homeostasis model assessment of insulin resistance; IP‐10, interferon‐gamma‐induced protein‐10; LDH, lactate dehydrogenase; M2BPGi, Mac2 binding protein glucosylation isomer; PDGF‐BB, platelet‐derived growth factor‐BB; PIIIP, type III procollagen peptidel; TIMP1, tissue inhibitor of metalloproteinases‐1.

Ultrasonography showed a bright liver and liver–kidney contrast, indicating the presence of fatty liver. A marked increase was observed in hepatic steatosis indices such as the hepatic steatosis index and non‐alcoholic fatty liver disease‐liver fat score (Table 1). Although we did not perform a hepatic biopsy, the degree of hepatic fibrosis was evaluated using noninvasive tests. Although the platelet count and APRI were within normal limits, an increase in the values of various noninvasive test parameters was observed. Particularly, the FIB‐4 index and serum M2BPGi levels were 1.96 and 1.19 C.O.I., respectively. Furthermore, the HFS and ELF scores were 0.4 and 10.8, respectively. Based on these findings, the stage of hepatic fibrosis was determined as F2.

An orthopedic disease rendered the patient unable to perform high‐intensity and long‐duration exercises. Therefore, she was instructed to perform low‐intensity short‐duration exercises. The exercise program included warm‐up (stepping as dynamic stretching), four types of resistance training (morning strength exercise, lateral pulldown exercise with a towel, squat exercise, and calf‐raise exercise), and stretching (the triceps femoris muscle). The exercise program mainly targeted the trunk and lower limbs. She repeated each of these exercises 10 times. The exercise intensity was less than three metabolic equivalents, and the total exercise duration was approximately 10 min. The exercise program was executed once daily for 60 weeks. Except for performing this exercise, there was no change in the lifestyle habits and medications of the patient.

This patient showed 5.7% body weight reduction 16 weeks after initiating this low‐intensity 10‐min exercise program. The weight loss continued until 60 weeks, when the patient achieved 9.1% body weight reduction (Table 1). A marked decrease was observed in the HOMA‐IR value, serum triglyceride level, and NAFLD‐liver fat score 16 weeks after initiating this low‐intensity 10‐min exercise program for 60 weeks (Table 1). At 16 weeks after initiating the exercise program, a marked decrease was observed in the Hepamet fibrosis and ELF scores (Table 1). Subsequently, the FIB‐4 index and the M2BPGi and autotaxin serum levels decreased to the reference values 60 weeks after initiating the program (Table 1). Although the FibroScan‐AST (FAST) score could not be evaluated by accumulated subcutaneous fat at 0 week, her FAST score indicated a high risk for nonalcoholic steatohepatitis (NASH) 16 weeks after initiating the exercise program. The FAST score decreased to a low risk for NASH 33 weeks after initiating the exercise program and lasted until 60 weeks (Table 1).

We investigated the changes in 48 chemokines and cytokines using a multiplex analysis. Of these, the serum G‐CSF level was markedly increased with oscillation, and the serum IP‐10 and PDGF‐BB levels were markedly decreased (Table 1). The other results showed in Table S1. The knee pain was stable, and no adverse events were observed throughout the observational period.

## Discussion

We report a case of NAFLD with elevated hepatic fibrosis indices, which was improved by a 60‐week low‐intensity 10‐min resistance exercise program. Additionally, we showed that our exercise program affected the serum G‐CSF, IP‐10, and PDGF‐BB levels in a patient with NAFLD.

Our exercise program improved the results of noninvasive tests for hepatic fibrosis. O'Gorman et al. reported that 12 weeks of moderate to vigorous aerobic exercise improved hepatic histology, such as hepatocyte ballooning and hepatic fibrosis.^1^ Thus, these studies showed that 12 weeks of moderate‐ to high‐intensity aerobic exercise could improve hepatic fibrosis. However, the effects of low‐intensity short‐duration exercise on hepatic fibrosis remain unclear.

To our knowledge, this is the first report to show that a low‐intensity 10‐min resistance exercise program could improve the outcomes of noninvasive tests for hepatic fibrosis, along with a rapid improvement in insulin resistance. Additionally, among various noninvasive tests, the Hepamet score and ELF score improved quickly in response to exercise, indicating the usefulness of the treatment for hepatic fibrosis. Although the mechanisms remain unclear, Takahashi et al. demonstrated that simple resistance exercises improve the parameters of metabolic syndrome, including insulin resistance with a reduction in serum cytokeratin 18 and fibroblast growth factor 21 levels, in patients with NAFLD.^7^ Insulin resistance is a crucial factor affecting hepatic fibrosis.^7^ This study suggests that our exercise program improves hepatic fibrosis by ameliorating insulin resistance.

Another possible mechanism is weight reduction. In general, 10% reduction in body weight is required to improve hepatic fibrosis^8^, ^9^ The patient showed a 5.7% reduction in body weight after the initiation exercise at 16 weeks. The weight loss lasted for 60 weeks, when the patient showed a 9.1% reduction in body weight. Taken together, both improvement of insulin resistance and weight reduction might contribute the improvement of liver fibrosis.

We investigated the effects of exercise on chemokines. The exercise increased the serum G‐CSF level and decreased the serum IP‐10 and PGFF‐BB levels. Mooren et al. reported an increase in the plasma G‐CSF level after resistance exercise in healthy men.^10^ G‐CSF promotes regeneration in the fibrotic liver through upregulation of the mobilization and proliferation of hepatic progenitor cells. Mendelson et al. reported that 12 weeks of aerobic exercise improved insulin resistance with a decrease in the serum IP‐10 level in obese participants.^11^ Moreover, IP‐10 positively correlates with hepatic fibrosis in patients with NAFLD. IP‐10 binds to C‐X‐C motif chemokine receptor 3, leading to hepatic inflammation through T‐lymphocyte activation. Zalm et al. reported that long‐term exercise decreased the serum PDGF‐BB level in obese participants.^12^ PDGF‐BB activates hepatic stellate cells to release collagen to promote hepatic fibrosis. Overall, these alterations in cytokines/chemokines may contribute to the amelioration of hepatic fibrosis after exercise.

## Conclusion

We encountered a case of NAFLD with elevated hepatic fibrosis indices, which were improved by a 60‐week low‐intensity 10‐min resistance daily exercise program. Additionally, our exercise program altered the serum G‐CSF, IP‐10, and PGFF‐BB levels, which are associated with hepatic fibrosis. Thus, a low‐intensity 10‐min resistance exercise daily may be beneficial for NAFLD patients with hepatic fibrosis.

## Patient consent statement

We obtained informed consent from the patient to use her medical data for this manuscript.

## Supporting information


**Table S1.** Changes in cytokine 48‐plex examination test results after initiating a low‐intensity, short‐duration exercise program.
