# Supplementary material for: A low‐intensity 10‐min resistance exercise program that ameliorated hepatic fibrosis indices and altered G‐CSF/IP‐10/PDGF‐BB in a patient with nonalcoholic fatty liver disease: A case report
Source: JGH Open. 2023 Feb 20;7(3):231–4. doi: 10.1002/jgh3.12876 (PMC10037032; doi:10.1002/jgh3.12876)
Supplement: Supplementary file 1 — Table S1. Changes in cytokine 48‐plex examination test results after initiating a low‐intensity, short‐duration exercise program. [file JGH3-7-231-s001.docx]

Supplementary Table 1. Changes in cytokine 48-plex examination test results after initiating a low-intensity short-duration exercise program

| Parameter | 0 | 16 | 33 | 60 |
| --- | --- | --- | --- | --- |
| Granulocyte-macrophage colony-stimulating factor (pg/mL) | N.D. | 0.68 | N.D. | N.D. |
| Growth related oncogene-a　(pg/mL) | 606 | 719.28 | 605.26 | 669.13 |
| Hepatocyte growth factor (pg/mL) | 599.3 | 729.27 | 619.36 | 699.81 |
| Interferon-a2 (pg/mL) | N.D. | 9.34 | 42 | 33 |
| Interleukin-1b (pg/mL) | 1.54 | 4.6 | 1.93 | 2.97 |
| Interleukin-6 (pg/mL) | 2.97 | 9.97 | 2.57 | 3.1 |
| Interleukin-7 (pg/mL) | *3.23 | 4.55 | N.D. | N.D. |
| Interleukin-8 (pg/mL) | 15.07 | 140.64 | 41.57 | 87.73 |
| Interleukin-9 (pg/mL) | 463.51 | 430.22 | 443.5 | 459.83 |
| Leukemia inhibitory factor (pg/mL) | 18.41 | 52.88 | 28.35 | 45.21 |
| Macrophage colony-stimulating factor (pg/mL) | 11.44 | 15.96 | 9.68 | 11.55 |
| Modified coronally advanced flap (pg/mL) | 63.37 | 59.89 | 64.25 | 59.23 |
| Monocyte chemotactic protein-3　 (pg/mL) | 0.28 | 1.45 | 0.51 | 0.97 |
| Macrophage migration inhibitory factor (pg/mL) | 347.16 | 939.63 | 576.02 | 611.27 |
| Monokine induced by interferon-gamma (pg/mL) | 424.23 | 1165.63 | 508.37 | 346.29 |
| Macrophage inflammatory protein-1a (pg/mL) | 4.22 | 115.19 | 32.02 | 83.21 |
| Macrophage inflammatory protein-1b (pg/mL) | 231.72 | 388.63 | 253.5 | 322.97 |
| Regulated on activation normal T-cell expressed and secreted genes (pg/mL) | 7950.59 | 8712.66 | 7856.36 | 7439.5 |
| Stem cell factor (pg/mL) | 32.43 | 42.04 | 28.79 | 34.45 |
| Stem cell growth factor-b (pg/mL) | 163709.78 | 184034.58 | 154039.27 | 147203.44 |
| Stromal cell-derived factor-1a (pg/mL) | 1695.05 | 1882.29 | 1700.27 | 1542.15 |
| Tumor necrosis factor alpha (pg/mL) | 53.89 | 163.67 | 80.18 | 126.15 |
| Tumor necrosis factor beta (pg/mL) | 625.02 | 623.42 | 601.2 | 596.27 |
| TNF-related apoptosis-inducing ligand (pg/mL) | 57.25 | 76.18 | 56.08 | 63.28 |
| Vascular endothelial growth factor (pg/mL) | N.D. | 198.05 | N.D. | N.D. |
| Beta nerve growth factor (pg/mL) | 10.48 | 17.42 | 7.93 | 11.33 |

Abbreviations: N.D., Not detected
